# Virtual covariance matrix reconstruction-based adaptive beamforming for small aperture array

**DOI:** 10.1371/journal.pone.0293012

**Published:** 2023-10-19

**Authors:** Lin Chang, Hao Zhang, Hua Yang, Tingting Lv, Ning Tang

**Affiliations:** Faculty of Information Science and Engineering, Ocean University of China, Qingdao, China; The Hong Kong Polytechnic University, HONG KONG

## Abstract

Recently, many robust adaptive beamforming (RAB) algorithms have been proposed to improve beamforming performance when model mismatches occur. For a uniform linear array, a larger aperture array can obtain higher array gain for beamforming when the inter-sensor spacing is fixed. However, only the small aperture array can be used in the equipment limited by platform installation space, significantly weakening beamforming output performance. This paper proposes two beamforming methods for improving beamforming output in small aperture sensor arrays. The first method employs an integration algorithm that combines angular sector and gradient vector search to reconstruct the interference covariance matrix (ICM). Then, the interference-plus-noise covariance matrix (INCM) is reconstructed combined with the estimated noise power. The INCM and ICM are used to optimize the desired signal steering vector (SV) by solving a quadratically constrained quadratic programming (QCQP) problem. The second method proposes a beamforming algorithm based on a virtual extended array to increase the degree of freedom of the beamformer. First, the virtual conjugated array element is designed based on the structural characteristics of a uniform linear array, and received data at the virtual array element are obtained using a linear prediction method. Then, the extended INCM is reconstructed, and the desired signal SV is optimized using an algorithm similar to the actual array. The simulation results demonstrate the effectiveness of the proposed methods under different conditions.

## Introduction

Adaptive beamforming technology has also been known as spatial filtering anti-interference technology, and there have been numerous research breakthroughs in this field in recent years [1–3]. The principle of this technology is to process sample data received by each sensor according to certain beamforming criteria and using various algorithms. After performing the weighted superposition of data received by each array element, the main lobe of the beam is aligned with the signal of interest (SOI), and a null is formed in the direction of the interference signal to suppress the interference, which effectively improves the system’s output performance. Because most methods based on this technology require prior information on the incoming direction of the desired signal, they have been frequently combined with the high-resolution direction of arrival (DOA) estimation algorithms [4, 5] to improve an antenna array system’s interference suppression ability and output performance. A minimum variance distortionless response (MVDR) beamformer can maximize the output signal-to-interference-plus-noise ratio (SINR) under the ideal condition that the SOI steering vector (SV) and interference-plus-noise covariance matrix (INCM) are known [6, 7]. However, the effect of adaptive beamforming can significantly decrease in many practical applications compared with the ideal situation due to the presence of SOI in the sample covariance matrix (SCM), array geometry errors, and DOA mismatch.

Recently, several robust adaptive beamforming (RAB) techniques have been proposed to reduce the effect of model mismatches and improve the robustness of beamforming. For instance, the eigenspace-based method [8–11] can help to improve the robustness of beamforming algorithms. However, noise pollution at a low signal-to-noise ratio (SNR) can degrade performance to a certain extent; also, the determination result of the subspace dimension can affect the output of this method. Diagonal loading (DL) [12–14] has been a common method for increasing the robustness of beamforming. However, the setting of the diagonal loading level determines the performance of beamformers. Although the adaptive diagonal loading algorithm [15, 16] can obtain a loading level adaptively, it is challenging to maintain robustness in a wide range of input SNR. In [17–20], the uncertainty set constraint technique was proposed to attain higher performance. The aforementioned methods are effective at improving the output SINR under certain error conditions.

Further, to minimize the desired signal’s impact on adaptive beamformers, the INCM reconstruction method [21, 22] was proposed to improve output performance by weakening the SOI in the SCM. In [21], the authors first proposed the idea of INCM reconstruction, where the INCM was reconstructed by using the Capon power spectrum integrated over an interference-plus-noise sector region, and the SV of the desired signal was optimized by solving a quadratically constrained quadratic programming (QCQP) problem. In [23], a low-complexity INCM reconstruction method based on spatial power spectrum sampling was developed. The method in [24] applied maximum entropy power spectrum to reconstruct the INCM by integrating over the angular sector of interference-plus-noise as well as the desired signal region. In [25], an effective RAB method was developed by reconstructing the INCM based on a combination of power method processing and spatial spectrum matching. In [26], the distributed digital subarray antennas were used to form a contiguous virtual array after the gaps between the subarrays were filled with virtual array elements. Then, the INCM of the contiguous virtual array was reconstructed. [27] proposed an extended INCM reconstruction method based on linear prediction to generate virtual sensor data and extend array aperture. In addition, combining the INCM reconstruction method and the coprime array [28, 29] can further enhance the output performance of beamforming.

What’s more, the array aperture size also affects the beamforming performance. Under particular experimental conditions, such as small detection or communication devices, the spatial aperture of an array can be smaller than the wavelengths involved [30]. For linear arrays, the array aperture size is related to both the interval of elements and the number of elements. When the number of array elements is fixed while the interval of elements decreases, or when the interval of elements is fixed while the number of elements decreases, the array aperture will decrease, so the beamforming performance will decline. In order to improve the output performance of beamforming algorithms on small aperture arrays, this paper proposes two RAB approaches based on the INCM-reconstructed method. In the first proposed method, a RAB algorithm based on an actual array is proposed. This algorithm combines the angular sector integration method and line searching integration method along the gradient vector to reconstruct the interference covariance matrix (ICM), and the INCM is constructed by combining the obtained ICM with the estimated noise covariance matrix. Then, the reconstructed ICM and INCM are used to optimize the desired signal SV by solving the QCQP problem. In the second method, a RAB algorithm based on a virtual extended array is proposed. First, the virtual conjugated element is generated according to the structural characteristics of a uniform linear array, and data received at the virtual array element are predicted using the linear prediction method. Further, the INCM of the extended array is reconstructed using the ICM reconstruction algorithm in the first method, and the desired signal SV is optimized. The main contributions of this paper include:

1: Two beamforming algorithms for small aperture arrays are proposed. The first proposed method combines the angular sector integration method and line searching integration method along the gradient vector to reconstruct the ICM, which achieves well performance in different conditions.

2: The second proposed method utilizes the linear prediction method to get a virtual extended array. Based on the ICM reconstruction and the desired signal SV optimization algorithm in the first method, the virtual element is used to expand the array’s aperture and improve the degree of freedom of the array.

The remainder of this paper is organized as follows. Section II describes the signal model and provides necessary background information about adaptive beamforming. Section III introduces the proposed RAB methods. Section IV presents simulation results. Finally, Section V concludes this paper.

## Signal model

Consider a uniform linear array (ULA) with *M* elements that receive *Q* far-field uncorrelated narrowband signals, including one desired signal and *Q* − 1 interference signals. Then, the *k*th array received signal can be expressed as follows:

$$\begin{array}{c} \text{x}\left(k\right)={\text{a}}_{1}s_{1}\left(k\right)+\sum_{q=2}^{Q}{\text{a}}_{q}s_{q}\left(k\right)+\text{n}\left(k\right) \end{array}$$ (1)

where the waveform of signal *s*_1_ is regarded as the SOI, and **a**_1_ is the corresponding SV; *s*_*q*_ and **a**_*q*_ denote the waveform and SV of the *q*th interference signal, respectively; **n** is the additive white Gaussian noise.

An MVDR beamformer obtains an optimal weighting vector by minimizing interference and noise power without distorting the SOI, which can be expressed as follows:

$$\begin{array}{c} \underset{\text{w}}{\text{min}}\;{\text{w}}^{H}{\text{R}}_{in}\text{w}\;\text{s.t.}\;{\text{w}}^{H}{\text{a}}_{1}=1 \end{array}$$ (2)

where **R**_*in*_ is the INCM, which is given by:

$$\begin{array}{c} \begin{array}{cc} {\text{R}}_{in} & =\sum_{q=2}^{Q}\sigma_{q}^{2}{\text{a}}_{q}{{\text{a}}_{q}}^{H}+\sigma_{n}^{2}{\text{I}}_{M}\\ & ={\text{R}}_{i}+\sigma_{n}^{2}{\text{I}}_{M} \end{array} \end{array}$$ (3)

where **R**_*i*_ and **I**_*M*_ represent the ICM and identity matrix, respectively; $\sigma_{q}^{2}$ and $\sigma_{n}^{2}$ are the powers of the *q*th interference signal and white Gaussian noise, respectively.

The solution to the optimal weighting vector is given by:

$$\begin{array}{c} {\text{w}}_{opt}=\frac{{\text{R}}_{in}^{-1}{\text{a}}_{1}}{{{\text{a}}_{1}}^{H}{\text{R}}_{in}^{-1}{\text{a}}_{1}} \end{array}$$ (4)

Because the actual INCM and SV are unavailable in real applications, the INCM can be replaced in two ways. One is to use the SCM $\hat{\text{R}}$, which can be written as follows:

$$\begin{array}{c} \hat{\text{R}}=\frac{1}{K}\sum_{k=1}^{K}\text{x}\left(k\right){\text{x}}^{H}\left(k\right) \end{array}$$ (5)

where *K* is the number of snapshots.

Another way is to reconstruct an INCM based on the available information.

The output of the beamformer is given by:

$$\begin{array}{c} y\left(k\right)={\text{w}}^{H}\text{x}\left(k\right) \end{array}$$ (6)

In the remainder of this paper, the hypothetical DOA of the SOI and *q*th interference are respectively denoted by ${\hat{\theta }}_{1}$ and ${\hat{\theta }}_{q}$, where *q* = 2, 3, …, *Q*. The desired signal SV obtained using array geometry and hypothetical DOA information is denoted by ${\hat{\text{a}}}_{1}$, and the *q*th interference SV is denoted by ${\hat{\text{a}}}_{q}$.

## Proposed methods

Generally, the gain of an array system is proportional to the array aperture size. As the array aperture size decreases, the array system’s gain will also decrease. The output performance of small aperture arrays can be improved by designing more robust beamformers or extending arrays with virtual elements.

### Beamforming algorithm based on actual array

The covariance matrix reconstruction method, which uses a spatial power spectrum integral over a particular sector as the estimate of the matrix, can effectively improve the robustness of beamforming. Inspired by this, the prposed1 method reconstructs the ICM over an angular sector and a gradient vector set. The estimated noise power is calculated based on the relationship between residual noise and actual noise. Based on the obtained INCM, the nominal SV of the desired signal can be optimized by solving a QCQP problem.

#### ICM reconstruction

In addition to the method defined by Eq (5), a real INCM can also be replaced by reconstructing the INCM. The integral algorithm in the angle region of a signal has a strong robustness to the direction error and can reconstruct the ICM by integrating the Capon space spectrum in the angular sector of the interference, which can be expressed as follows:

$$\begin{array}{c} {\hat{\text{R}}}_{dq}=\int_{\Theta_{q}}\hat{P}\left(\theta \right)\hat{\text{a}}\left(\theta \right){\hat{\text{a}}}^{H}\left(\theta \right)d\theta \end{array}$$ (7)

where Θ_*q*_ represents the angular sector of the *q*th interference signal, and $\hat{P}\left(\theta \right)$ is the Capon spectrum in the *θ* direction, which can be expressed by [31]:

$$\begin{array}{c} \hat{P}\left(\theta \right)=\frac{1}{{\hat{\text{a}}}^{H}\left(\theta \right){\hat{\text{R}}}^{-1}\hat{\text{a}}\left(\theta \right)} \end{array}$$ (8)

Then, the ICM reconstruction in Θ_*q*_ can be approximately performed using a discrete sum method as follows:

$$\begin{array}{c} {\hat{\text{R}}}_{dq}\approx \sum_{l=1}^{L}\frac{\hat{\text{a}}\left(\theta_{l}\right){\hat{\text{a}}}^{H}\left(\theta_{l}\right)}{{\hat{\text{a}}}^{H}\left(\theta_{l}\right){\hat{\text{R}}}^{-1}\hat{\text{a}}\left(\theta_{l}\right)}△\theta \end{array}$$ (9)

where *θ*_*l*_, *l* = 1, 2, …, *L* is the sample points in Θ_*q*_, and *L* represents the number of discrete sample points.

The basic idea of reconstructing the ICM by Eq (9) is to use the Capon power spectrum integral in the angular sector where the interference is located. The Capon power spectrum is mainly distributed in the actual signal SV [32]. In addition, it can be considered that multiple virtual interference signals are added near the interference estimation direction ${\hat{\theta }}_{q}$. Although this reconstruction method can overcome the effect of direction estimation error on the calculation result to a certain extent, the acquisition of $\hat{P}\left(\theta \right)$ and $\hat{\text{a}}\left(\theta \right)$ requires accurate array calibration. If other array errors exist, the ICM reconstruction by Eq (9) will lead to not ideal results. The algorithm presented in [32] adopts a volume integral method for the INCM reconstruction to reduce the sensitivity to array calibration error. However, the number of discretization points in the integration region of [32] is two to the power of *M*. When the number of matrix elements is large, the number of discretization points will be too large to calculate.

Further, to address other errors and decrease the number of discretization points in the integration region, the ICM reconstruction along the gradient direction from ${\hat{\text{a}}}_{q}$ is proposed. The basic idea of this method is that there can be multiple contour vectors with the same Capon power value near an actual signal SV, and ${\hat{\text{a}}}_{q}$ is one of them. Therefore, if several nominal SVs are selected in the small range near ${\hat{\theta }}_{q}$ to form a hyperplane, the estimated SV ${\hat{\text{a}}}_{q}$ can be regarded as a tangent point between the hyperplane and the contour line. Then, from the tangent point ${\hat{\text{a}}}_{q}$, the true SV can be found along the gradient direction perpendicular to the hyperplane [33]. Based on this inference, the ICM can be constructed from the tangent point ${\hat{\text{a}}}_{q}$ in the gradient direction using the line integral method.

Next, the gradient can be obtained by using the subspace-based method to reconstruct the scan matrix of the SV ${\hat{\text{a}}}_{q}$ in a certain range of directions as follows:

$$\begin{array}{c} {\text{H}}_{q}=\left[{\hat{\text{a}}}_{q}\left(\theta_{1}\right),{\hat{\text{a}}}_{q}\left(\theta_{2}\right),\ldots ,{\hat{\text{a}}}_{q}\left(\theta_{M-1}\right)\right] \end{array}$$ (10)

where [*θ*_1_, *θ*_2_, …, *θ*_*M*−1_] is a set of (*M* − 1) sample points uniformly distributed in Θ_*q*_.

Then the non-full rank matrix ${\text{H}}_{q}{\text{H}}_{q}^{H}$ can be constructed, and its eigendecomposition is performed as follows:

$$\begin{array}{c} \begin{array}{cc} {\text{H}}_{q}{\text{H}}_{q}^{H} & =\sum_{m=1}^{M}\gamma_{qm}{\text{b}}_{qm}{\text{b}}_{qm}^{H}\\ & =\sum_{m=1}^{M-1}\gamma_{qm}{\text{b}}_{qm}{\text{b}}_{qm}^{H}+\gamma_{qM}{\text{b}}_{qM}{\text{b}}_{qM}^{H} \end{array} \end{array}$$ (11)

where *γ*_*q*1_ ≥ *γ*_*q*2_ ≥ … ≥ *γ*_*qM*_ represents the eigenvalues, and **b**_*qm*_ is the eigenvector corresponding to *γ*_*qm*_.

Let **B**_*q*1_ = [**b**_*q*1_, **b**_*q*2_, …, **b**_*qM*−1_], which is a hyperplane space containing *M* − 1 eigenvectors corresponding to the maximum of (*M* − 1) eigenvalues. Since matrix ${\text{H}}_{q}{\text{H}}_{q}^{H}$ is not full rank, the eigenvectors corresponding to the first (*M* − 1) large eigenvalues are sufficient to span the space where the hyperplane is located. According to the property of the conjugate symmetric matrix, the eigenvector **b**_*qM*_ is orthogonal to the other eigenvectors. Further, **b**_*qM*_ is orthogonal to the hyperplane space, so it can be used as a gradient vector estimation of ${\hat{\text{a}}}_{q}$ [33].

The estimated nominal interference SV is adjusted by adding a scaled gradient vector:

$$\begin{array}{c} {\bar{\text{a}}}_{q}={\hat{\text{a}}}_{q}+\eta {\text{b}}_{qM} \end{array}$$ (12)

where *η* ∈ [−*ξ*, *ξ*].

Inspired by [33], the gradient-based reconstruction ICM can be expressed as a discrete sum of the adjusted SV, which can be expressed as follows:

$$\begin{array}{c} {\hat{\text{R}}}_{eq}\approx \sum_{\eta =-\xi }^{\xi }\frac{{\bar{\text{a}}}_{q}{\bar{\text{a}}}_{q}^{H}}{{\bar{\text{a}}}_{q}^{H}{\hat{\text{R}}}^{-1}{\bar{\text{a}}}_{q}}△\eta \end{array}$$ (13)

Combining Eqs (9) and (13), the final ICM can be reconstructed as follows:

$$\begin{array}{c} {\hat{\text{R}}}_{i}=\sum_{q=2}^{Q}\left({\hat{\text{R}}}_{dq}+{\hat{\text{R}}}_{eq}\right) \end{array}$$ (14)

#### Noise power estimation and SV optimization

Using the idea presented in [34], the noise power for the noise covariance matrix can be estimated as follows:

$$\begin{array}{c} {\hat{\sigma }}_{n}^{2}=M{\bar{\sigma }}_{n}^{2} \end{array}$$ (15)

where ${\bar{\sigma }}_{n}^{2}$ is the residual noise power, which is computed by:

$$\begin{array}{c} {\bar{\sigma }}_{n}^{2}=\frac{1}{J}\sum_{j=1}^{J}\frac{1}{{\hat{\text{a}}}^{H}\left(\theta_{j}\right){\hat{\text{R}}}^{-1}\hat{\text{a}}\left(\theta_{j}\right)} \end{array}$$ (16)

where *θ*_*j*_ is a discrete sample point in the noise region Θ_*n*_, and *J* is the number of sample points.

Then, the reconstructed INCM is given by:

$$\begin{array}{c} {\hat{\text{R}}}_{in}={\hat{\text{R}}}_{i}+{\hat{\sigma }}_{n}^{2}{\text{I}}_{M} \end{array}$$ (17)

The desired signal’s SV can be optimized by adopting the idea of maximizing the beamformer’s output power [21]. In addition, to prevent the estimated desired SV from converging to other interference regions, the ICMs obtained in two different ways, by Eqs (9) and (13), are both considered when designing the constrain conditions:

$$\begin{array}{c} \begin{array}{cc} & \underset{v_{s_{⊥}}}{\text{min}}\;{\left({\hat{\text{a}}}_{1}+{\text{v}}_{s_{⊥}}\right)}^{H}{\hat{\text{R}}}_{in}^{-1}\left({\hat{\text{a}}}_{1}+{\text{v}}_{s_{⊥}}\right)\\ & s.t.\;{\hat{\text{a}}}_{1}{\text{v}}_{s⊥}=0\\ & \;{\left({\hat{\text{a}}}_{1}+{\text{v}}_{s_{⊥}}\right)}^{H}{\hat{\text{R}}}_{d}\left({\hat{\text{a}}}_{1}+{\text{v}}_{s_{⊥}}\right)⩽{{\hat{\text{a}}}_{1}}^{H}{\hat{\text{R}}}_{d}{\hat{\text{a}}}_{1}\\ & \;{\left({\hat{\text{a}}}_{1}+{\text{v}}_{s_{⊥}}\right)}^{H}{\hat{\text{R}}}_{e}\left({\hat{\text{a}}}_{1}+{\text{v}}_{s_{⊥}}\right)⩽{{\hat{\text{a}}}_{1}}^{H}{\hat{\text{R}}}_{e}{\hat{\text{a}}}_{1} \end{array} \end{array}$$ (18)

where ${\hat{\text{R}}}_{d}=\sum_{q=2}^{Q}{\hat{\text{R}}}_{dq}+{\hat{\sigma }}_{n}^{2}{\text{I}}_{M}$, and ${\hat{\text{R}}}_{e}=\sum_{q=2}^{Q}{\hat{\text{R}}}_{eq}+{\hat{\sigma }}_{n}^{2}{\text{I}}_{M}$; ${\text{v}}_{s_{⊥}}$ is one of the components of **v**_*s*_, and it is orthogonal to ${\hat{\text{a}}}_{1}$; **v**_*s*_ is the mismatch vector of ${\hat{\text{a}}}_{1}$, and it can be decomposed into two components, namely ${\text{v}}_{s_{\left|\right|}}$ and ${\text{v}}_{s_{⊥}}$; ${\text{v}}_{s_{\left|\right|}}$ is parallel to ${\hat{\text{a}}}_{1}$ and does not affect the beamforming quality. Therefore, ${\text{v}}_{s_{\left|\right|}}$ is not considered in Eq (18).

The optimization problem in Eq (18) represents a quadratically constrained quadratic programming (QCQP) problem and can be solved by a convex optimization toolbox. The optimized SV is expressed as ${\hat{\bar{\text{a}}}}_{1}={\hat{\text{a}}}_{1}+{\text{v}}_{s_{⊥}}$, and the weight vector obtained based on the actual array can be calculated by:

$$\begin{array}{c} \text{w}=\frac{{\hat{\text{R}}}_{in}^{-1}{\hat{\bar{\text{a}}}}_{1}}{{\hat{\bar{\text{a}}}}_{1}^{H}{\hat{\text{R}}}_{in}^{-1}{\hat{\bar{\text{a}}}}_{1}} \end{array}$$ (19)

### Beamforming algorithm based on virtual extended array

By using the real data received from an actual array, a virtual array can construct the received data at the virtual element to expand the array. The proposed2 method extends the ICM reconstruction method in proposed1 to the virtual extension array using the virtual element. Compared with the original actual array, the advantage of a virtual extended array is that it can increase the number of degrees of freedom.

#### Extended array data generation

**Fig 1:**
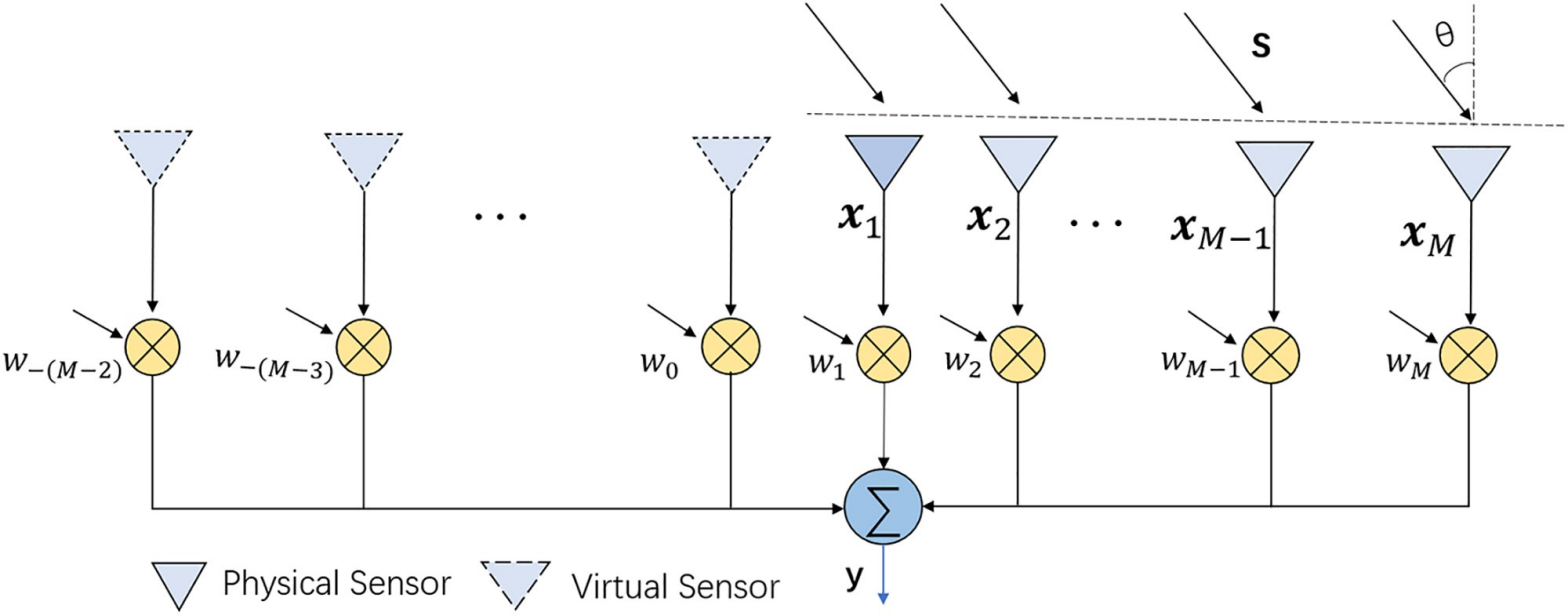
The structure of an extended sensor array.

A virtual extended array based on the MVDR method has three tasks to solve. The first task is to determine the expansion mode of a virtual array, that is, to determine the expansion array’s SV. The second task is to generate the virtual array’s received data, and the third task is to construct the INCM of the extended array. To extend the aperture and degree of freedom of an array, this study designs a virtual extended array using the conjugate symmetry of ULA [35]. The extended array is shown in Fig 1.

If the first element **x**_1_ of a real array is the point of symmetry, then the real and virtual elements are axially symmetric about **x**_1_. The number of sensor elements in an extended array is (2*M* − 1), of which (*M* − 1) elements are virtual elements. Assume that the signal SV of an actual array with DOA *θ* is $\hat{\text{a}}\left(\theta \right)={\left[1,e^{-j2\pi d\;\text{sin}\;\theta /\lambda },\ldots ,e^{-j2\pi (M-1)d\;\text{sin}\;\theta /\lambda }\right]}^{T}$; then, the SV of a virtual element is estimated to be expressed from right to left as follows:

$$\begin{array}{c} {\hat{\text{a}}}_{V}\left(\theta \right)={\left[e^{j2\pi d\;\text{sin}\;\theta /\lambda },\ldots ,e^{j2\pi (M-1)d\;\text{sin}\;\theta /\lambda }\right]}^{T} \end{array}$$ (20)

where λ represents the signal wavelength, and *d* is the spacing between adjacent sensors.

According to Eq (20), the SV of a virtual element is conjugate of the SV of an actual array. Thus, the extended-array SV estimation can be expressed as follows:

$$\begin{array}{c} \tilde{\text{a}}\left(\theta \right)={\left[e^{j2\pi (M-1)d\;\text{sin}\;\theta /\lambda },\ldots ,e^{j2\pi d\;\text{sin}\;\theta /\lambda },1,\ldots ,e^{-j2\pi (M-1)d\;\text{sin}\;\theta /\lambda }\right]}^{T} \end{array}$$ (21)

For the purpose of facilitating the subsequent calculation, the received data of a virtual extended array are expressed as follows:

$$\begin{array}{c} \tilde{\text{x}}\left(k\right)=\left[x_{-(M-2)}\left(k\right),x_{-(M-3)}\left(k\right),\ldots ,x_{0}\left(k\right),x_{1}\left(k\right),x_{2}\left(k\right),\ldots ,x_{M}\left(k\right)\right] \end{array}$$ (22)

Generally, except a DOA estimation algorithm can predict the DOA, information on each signal in an array is unknown, so the received data at a virtual element cannot be calculated directly by Eq (1). However, a linear prediction method can be used to generate a virtual array element’s data [27, 36]. For a conjugate virtual extended array, the *m*th forward prediction sensors can be estimated in an iterative way as follows:

$$\begin{array}{c} x_{-m}\left(k\right)={\left[x_{1-m}\left(k\right),\ldots ,x_{M-1-m}\left(k\right)\right]}^{T}\alpha \end{array}$$ (23)

where *m* = 0, 1, 2, …, *M* − 2, $\alpha ={\hat{\text{R}}}_{F}^{-1}{\text{d}}_{F}$ is the forward prediction coefficient, and ${\hat{\text{R}}}_{F}$ is the cross-correlation matrix of the input matrix for a linear system, which is expressed by:

$$\begin{array}{c} {\hat{\text{R}}}_{F}=\frac{1}{K}\sum_{k=1}^{K}{\text{x}}_{F}\left(k\right){\text{x}}_{F}^{H}\left(k\right) \end{array}$$ (24)

In the forward prediction method, the first real element is usually regarded as reference data, and the second to *M*th elements are considered the input matrix, which is expressed as **x**_*F*_(*k*) = [*x*_2_(*k*), …, *x*_*M*_(*k*)]^*T*^. Further, **d**_*F*_ is the cross-correlation between **x**_*F*_(*k*) and *x*_1_(*k*), and it is defined by:

$$\begin{array}{c} {\text{d}}_{F}=\frac{1}{K}\sum_{k=1}^{K}{\text{x}}_{F}\left(k\right)x_{1}^{H}\left(k\right) \end{array}$$ (25)

#### Extended INCM reconstruction and SV optimization

The extended SCM can be expressed as follows:

$$\begin{array}{c} \tilde{\text{R}}=\frac{1}{K}\sum_{k=1}^{K}\tilde{\text{x}}\left(k\right){\tilde{\text{x}}}^{H}\left(k\right) \end{array}$$ (26)

where $\tilde{\text{R}}$ is a semi-positive definite matrix with a dimension of (2*M* − 1) × (2*M* − 1), and its condition number is defined by:

$$\begin{array}{c} cond_{v}=\frac{{\tilde{\varepsilon }}_{max}}{{\tilde{\varepsilon }}_{min}} \end{array}$$ (27)

where ${\tilde{\varepsilon }}_{max}$ and ${\tilde{\varepsilon }}_{min}$ are the largest and the smallest eigenvalues of $\tilde{\text{R}}$, respectively.

It should be noted that when the number of virtual sensor array elements increases, the dimension and condition number of $\tilde{\text{R}}$ will increase accordingly. A high condition number causes numerical instability in matrix inversion, which can lead to errors in Capon spectrum estimation [27].

Since the condition number is related to the matrix’s eigenvalue, the eigenvalue can be changed using a diagonal loading algorithm to reduce the condition number. Assume that ${\tilde{\text{R}}}_{L}$ is the covariance matrix modified by applying a diagonal loading method on $\tilde{\text{R}}$; then, it holds that ${\tilde{\text{R}}}_{L}=\tilde{\text{R}}+\iota {\text{I}}_{2M-1}$. Therefore, a new condition number resulting from the diagonal loading level *ι* can be expressed as follows:

$$\begin{array}{c} cond_{v+\iota }=\frac{{\tilde{\varepsilon }}_{max}+\iota }{{\tilde{\varepsilon }}_{min}+\iota } \end{array}$$ (28)

To avoid relying on experience when designing *ι*, *cond*_*v*+*ι*_ can be limited by the condition number *cond*_*r*_ of $\hat{\text{R}}$. Further, define *cond*_*v*+*ι*_ ≤ *cond*_*r*_, such that:

$$\begin{array}{c} \frac{{\tilde{\varepsilon }}_{max}+\iota }{{\tilde{\varepsilon }}_{min}+\iota }\leq \frac{{\hat{\varepsilon }}_{max}}{{\hat{\varepsilon }}_{min}} \end{array}$$ (29)

where ${\hat{\varepsilon }}_{max}$ and ${\hat{\varepsilon }}_{min}$ are the maximum and minimum eigenvalues of $\hat{\text{R}}$, respectively. The value of a diagonal loading level needs to satisfy the following condition:

$$\begin{array}{c} \iota \geq \frac{{\hat{\varepsilon }}_{min}{\tilde{\varepsilon }}_{max}-{\hat{\varepsilon }}_{max}{\tilde{\varepsilon }}_{min}}{{\hat{\varepsilon }}_{max}-{\hat{\varepsilon }}_{min}} \end{array}$$ (30)

Therefore, the extended Capon spectrum in *θ* can be expressed as follows:

$$\begin{array}{c} \tilde{P}\left(\theta \right)=\frac{1}{{\tilde{\text{a}}}^{H}\left(\theta \right){\tilde{\text{R}}}_{L}^{-1}\tilde{\text{a}}\left(\theta \right)} \end{array}$$ (31)

According to Eq (7), the extended ICM related to the incident direction of the *q*th interference can be reconstructed as follows:

$$\begin{array}{c} {\tilde{\text{R}}}_{dq}\approx \sum_{l=1}^{L}\frac{\tilde{\text{a}}\left(\theta_{l}\right){\tilde{\text{a}}}^{H}\left(\theta_{l}\right)}{{\tilde{\text{a}}}^{H}\left(\theta_{l}\right){\tilde{\text{R}}}_{L}^{-1}\tilde{\text{a}}\left(\theta_{l}\right)}△\theta \end{array}$$ (32)

Similarly, the gradient-based extended ICM corresponding to the *q*th interference can be reconstructed as follows:

$$\begin{array}{c} {\tilde{\text{R}}}_{eq}\approx \sum_{\eta =-\xi }^{\xi }\frac{{\bar{\tilde{\text{a}}}}_{q}{\bar{\tilde{\text{a}}}}_{q}^{H}}{{\bar{\tilde{\text{a}}}}_{q}^{H}{\tilde{\text{R}}}_{L}^{-1}{\bar{\tilde{\text{a}}}}_{q}}△\eta \end{array}$$ (33)

where ${\bar{\tilde{\text{a}}}}_{q}={\tilde{\text{a}}}_{q}+\eta {\tilde{\text{b}}}_{qM}$, and ${\tilde{\text{b}}}_{qM}$ is the gradient vector estimation obtained based on the *q*th interference signal’s extended SV.

Combining Eqs (32) and (33), the final extended ICM can be reconstructed by:

$$\begin{array}{c} {\tilde{\text{R}}}_{i}=\sum_{q=2}^{Q}\left({\tilde{\text{R}}}_{dq}+{\tilde{\text{R}}}_{eq}\right) \end{array}$$ (34)

Furthermore, the estimated extended INCM can be calculated by:

$$\begin{array}{c} {\tilde{\text{R}}}_{in}={\tilde{\text{R}}}_{i}+{\hat{\sigma }}_{n}^{2}{\text{I}}_{2M-1} \end{array}$$ (35)

In this study, the extended SV of the desired signal is optimized using the method defined by Eq (18) as follows:

$$\begin{array}{c} \begin{array}{cc} & \underset{{\tilde{\text{v}}}_{s⊥}}{\text{min}}\;{\left({\tilde{\text{a}}}_{1}+{\tilde{\text{v}}}_{s_{⊥}}\right)}^{H}{\tilde{\text{R}}}_{in}^{-1}\left({\tilde{\text{a}}}_{1}+{\tilde{\text{v}}}_{s_{⊥}}\right)\\ & s.t.\;{\tilde{\text{a}}}_{1}{\tilde{\text{v}}}_{s⊥}=0\\ & \;{\left({\tilde{\text{a}}}_{1}+{\tilde{\text{v}}}_{s_{⊥}}\right)}^{H}{\tilde{\text{R}}}_{d}\left({\tilde{\text{a}}}_{1}+{\tilde{\text{v}}}_{s_{⊥}}\right)⩽{{\tilde{\text{a}}}_{1}}^{H}{\tilde{\text{R}}}_{d}{\tilde{\text{a}}}_{1}\\ & \;{\left({\tilde{\text{a}}}_{1}+{\tilde{\text{v}}}_{s_{⊥}}\right)}^{H}{\tilde{\text{R}}}_{e}\left({\tilde{\text{a}}}_{1}+{\tilde{\text{v}}}_{s_{⊥}}\right)⩽{{\tilde{\text{a}}}_{1}}^{H}{\tilde{\text{R}}}_{e}{\tilde{\text{a}}}_{1} \end{array} \end{array}$$ (36)

where ${\tilde{\text{R}}}_{d}=\sum_{q=2}^{Q}{\tilde{\text{R}}}_{dq}$, and ${\tilde{\text{R}}}_{e}=\sum_{q=2}^{Q}{\tilde{\text{R}}}_{eq}$; ${\tilde{\text{a}}}_{1}$ is the extended SV estimation of the desired signal; ${\tilde{\text{v}}}_{s⊥}$ is one of the components of ${\tilde{\text{v}}}_{s}$, and it is orthogonal to ${\tilde{\text{a}}}_{1}$; ${\tilde{\text{v}}}_{s}$ is the mismatch vector of ${\tilde{\text{a}}}_{1}$.

The convex optimization toolbox can also be used to solve the QCQP problem in Eq (36). The extended optimized SV is expressed as ${\tilde{\bar{\text{a}}}}_{1}={\tilde{\text{a}}}_{1}+{\tilde{\text{v}}}_{s_{⊥}}$, and the weight vector obtained based on the virtual extended array can be calculated by:

$$\begin{array}{c} \tilde{\text{w}}=\frac{{\tilde{\text{R}}}_{in}^{-1}{\tilde{\bar{\text{a}}}}_{1}}{{\tilde{\bar{\text{a}}}}_{1}^{H}{\tilde{\text{R}}}_{in}^{-1}{\tilde{\bar{\text{a}}}}_{1}} \end{array}$$ (37)

Finally, the corresponding extended array beamforming output is given by:

$$\begin{array}{c} \tilde{y}\left(k\right)={\tilde{\text{w}}}^{H}\tilde{\text{x}}\left(k\right) \end{array}$$ (38)

## Simulation results

In this study, a ULA with *M* = 10 omnidirectional sensors was used. There were four independent signals impinging from the directions of 5°, −30°, 30°, and 50°. It was supposed the first signal was the desired signal while the other signals were interference signals. The interference-to-noise ratio (INR) of the first interference signal was 10 dB, and the INR of the second and the third interference signals was 30 dB. The proposed beamformer was compared with the INCM reconstruction-based beamformer with volume integration (INCM-volume) [32], INCM reconstruction method based on simplified interference power estimation (INCM-Simplified) [37], the second INCM reconstruction method based on a subspace (INCM-subspace) [34], INCM reconstruction beamformer based on a local maximum of the Capon power (INCM-MCP) [33], INCM reconstruction method with subspace decomposition, steering vector estimation and correction [38] (INCM-SDC), the extended virtual method based on linear prediction [27] (LPV), and signal vector estimation [26] (SFV). In this section, the proposed beamforming method that uses Eq (19) to obtain the weight vector is referred to as proposed1, and the method that uses Eq (37) to determine the weight vector is denoted as proposed2.

The interference and SOI angular sector were $\Theta_{q}=\left[{\hat{\theta }}_{q}-5^{\circ },{\hat{\theta }}_{q}+5^{\circ }\right]$ and $\Theta_{s}=\left[{\hat{\theta }}_{1}-5^{\circ },{\hat{\theta }}_{1}+5^{\circ }\right]$, respectively; the complement sector of interference and SOI was defined as a noise region Θ_*n*_. A uniform sample angular interval 0.1° was used in all angular sectors. The number of SVs constructed for **H**_*q*_ in the actual experiment was the same as the number of samples in Θ_*q*_ to achieve better performance. The number of sampling points in Θ_*int*_ was *I* = 40 for the INCM-volume method. The number of dominant eigenvectors was set as seven in the INCM-subspace method. The parameter *ξ* in the INCM-MCP and proposed methods was 0.1 with an interval of 0.01. The number of virtual elements in the LPV was set to 20. The virtual element in the SFV was obtained by using the virtual conjugate extended array.

In the SINR calculation, the INCM of all virtual array methods was obtained using the data received by the real extended array. When the SNR of the SOI changed, the number of snapshots was fixed to *K* = 1, 000. When the number of snapshots varied, the SNR was fixed to 10 dB. The sample data used in all simulations contained the SOI at all times, and all the results are the average of 200 Monte Carlo runs. The QCQP problem was solved using the CVX [39].

### Exactly known signal steering vector

**Fig 2:**
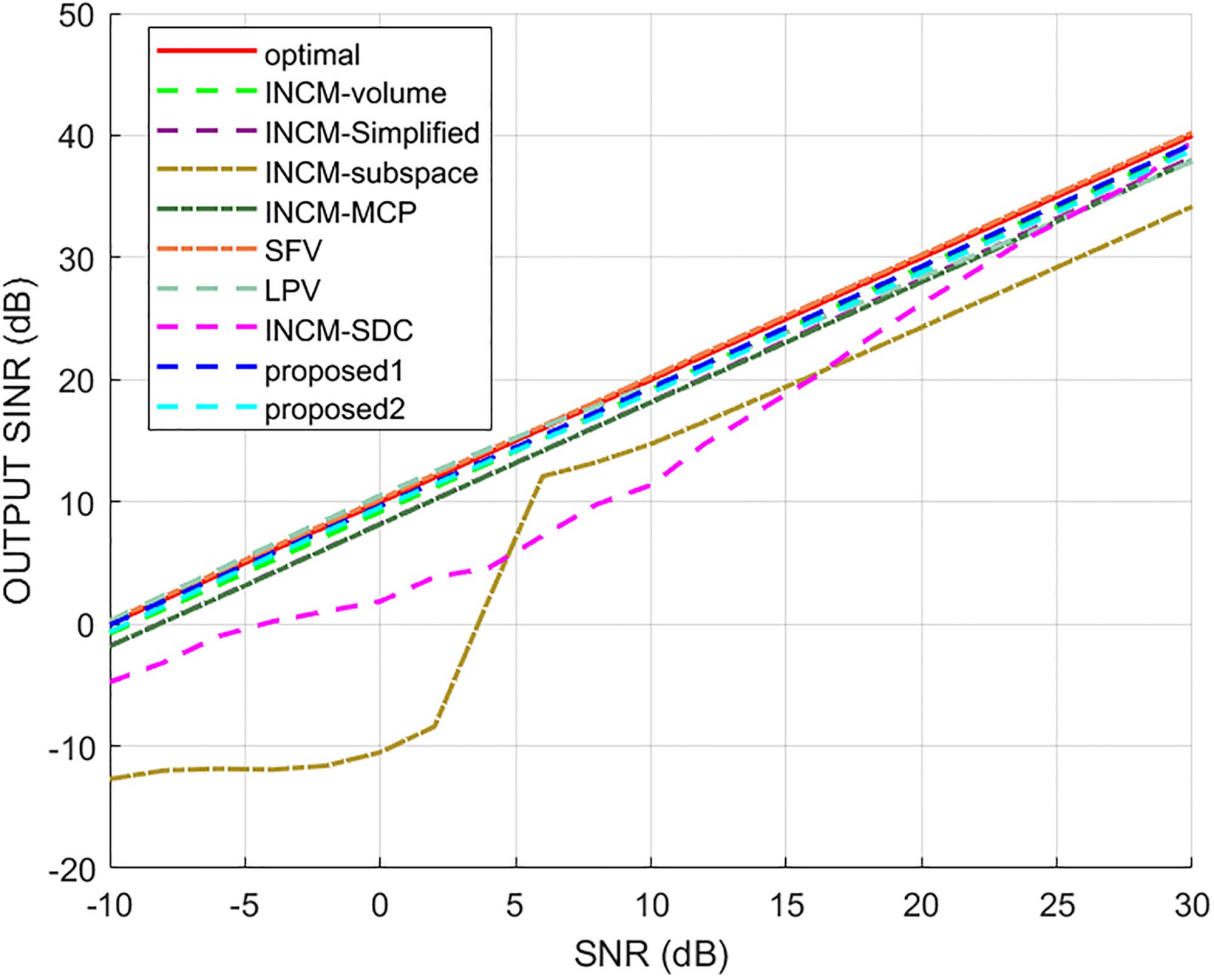
The output SINR versus the input SNR.

**Fig 3:**
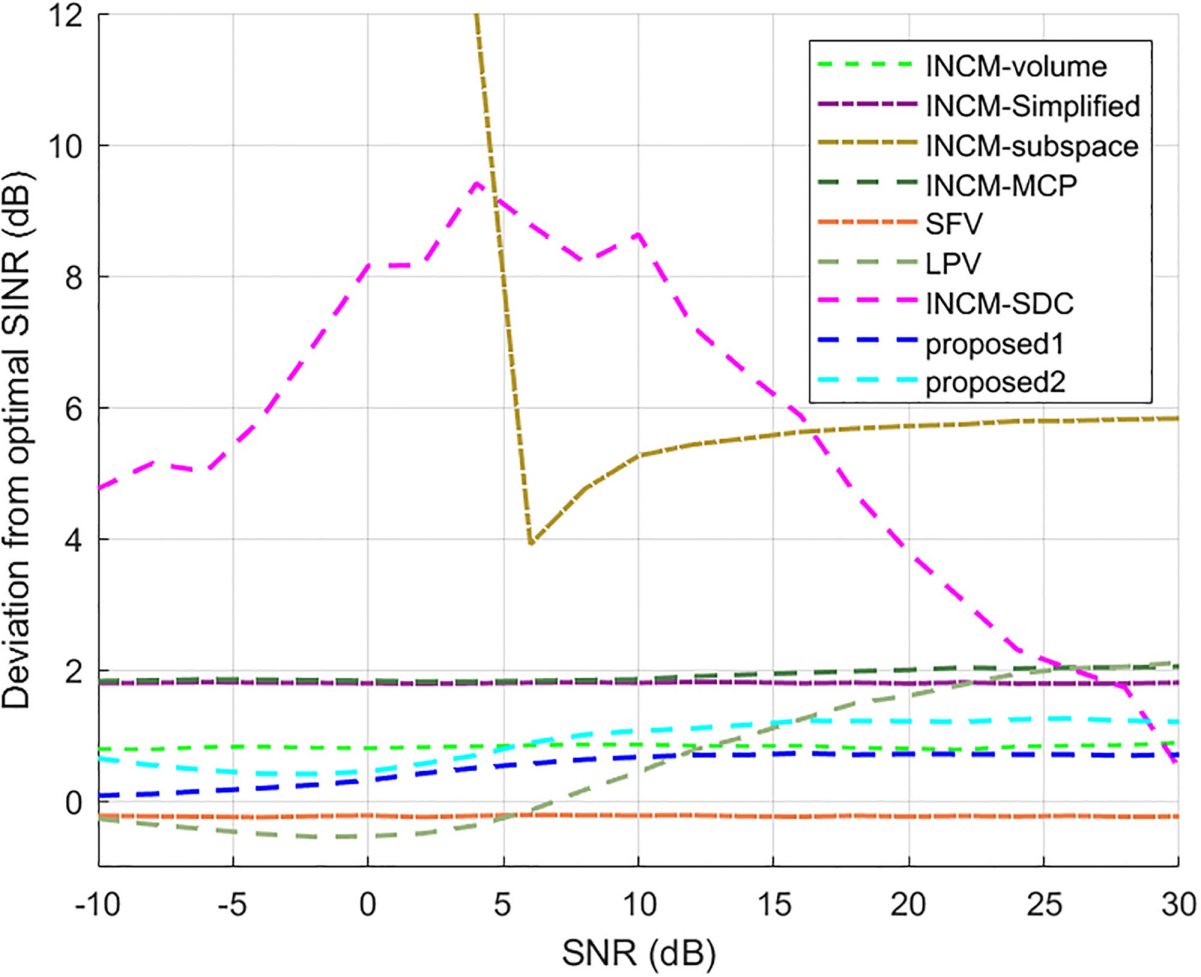
Deviations from the optimal SINR versus the SNR value.

**Fig 4:**
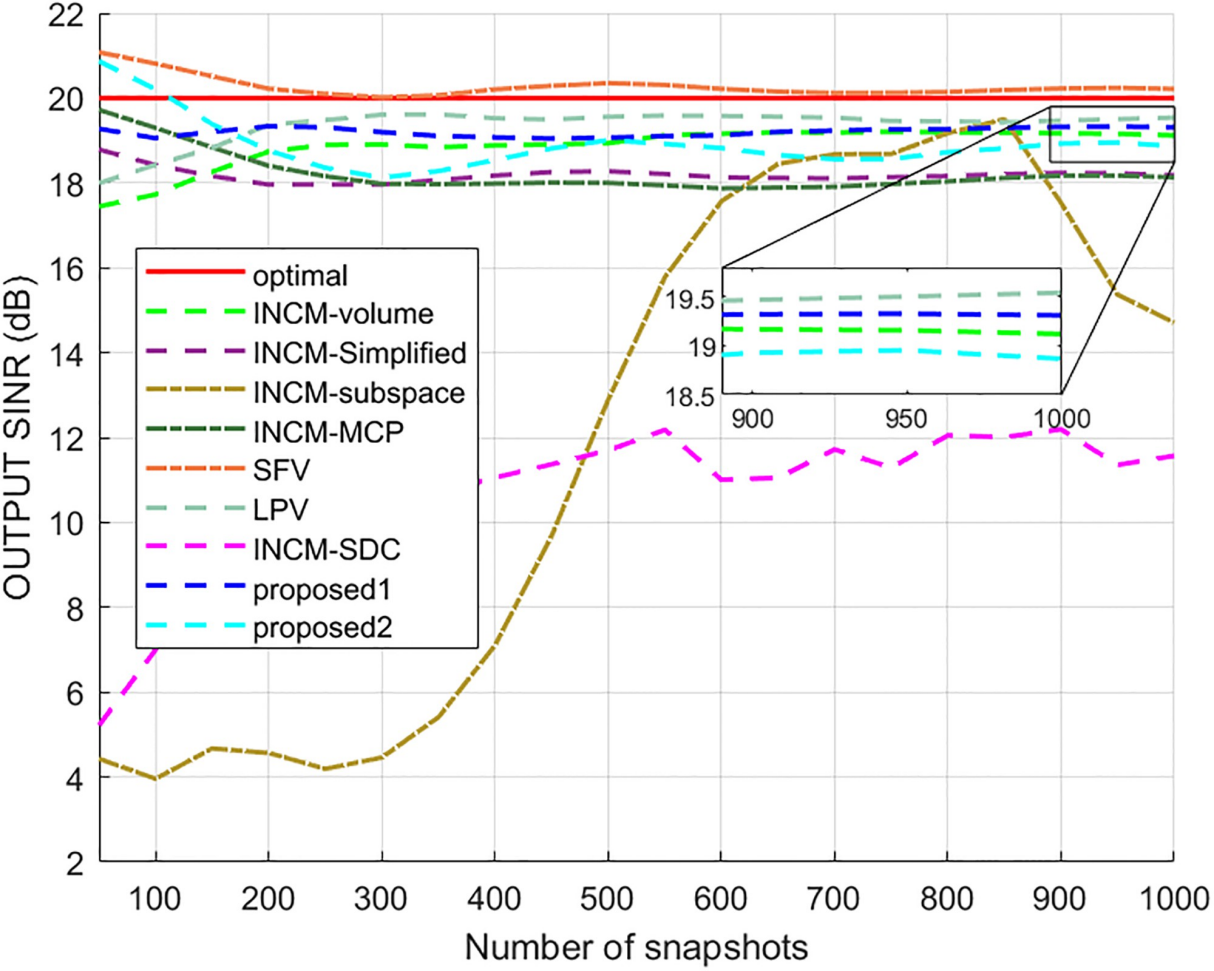
The output SINR versus the number of snapshots.

This example considered the situation where the exact steering vector was known. Fig 2 displays the output SINR of the proposed methods versus the SNR, and Fig 3 shows the deviations between all tested methods and the optimal SINR. The performance of the SFV beamformer based on a virtual extended array was higher than the proposed algorithms and optimal values in this test. This is because there is no error in the SV, and when the information of the SV is completely known, this method can obtain more accurate incident signals. Then the ICM obtained by combining the incident signals with the known extended SV will be close to the ICM of the real extended array. The output of the extended virtual array beamformer LPV was also higher than the optimal values when the SNR was less than 6 dB. However, the output performance of LPV deteriorates as the input SNR increases. The output SINR of the tested beamformers versus the number of snapshots is presented in Fig 4, where it can be seen that the output SINR results of the SFV and LPV were better than those of the proposed methods and other tested beamformers.

### Mismatch caused by look direction error

**Fig 5:**
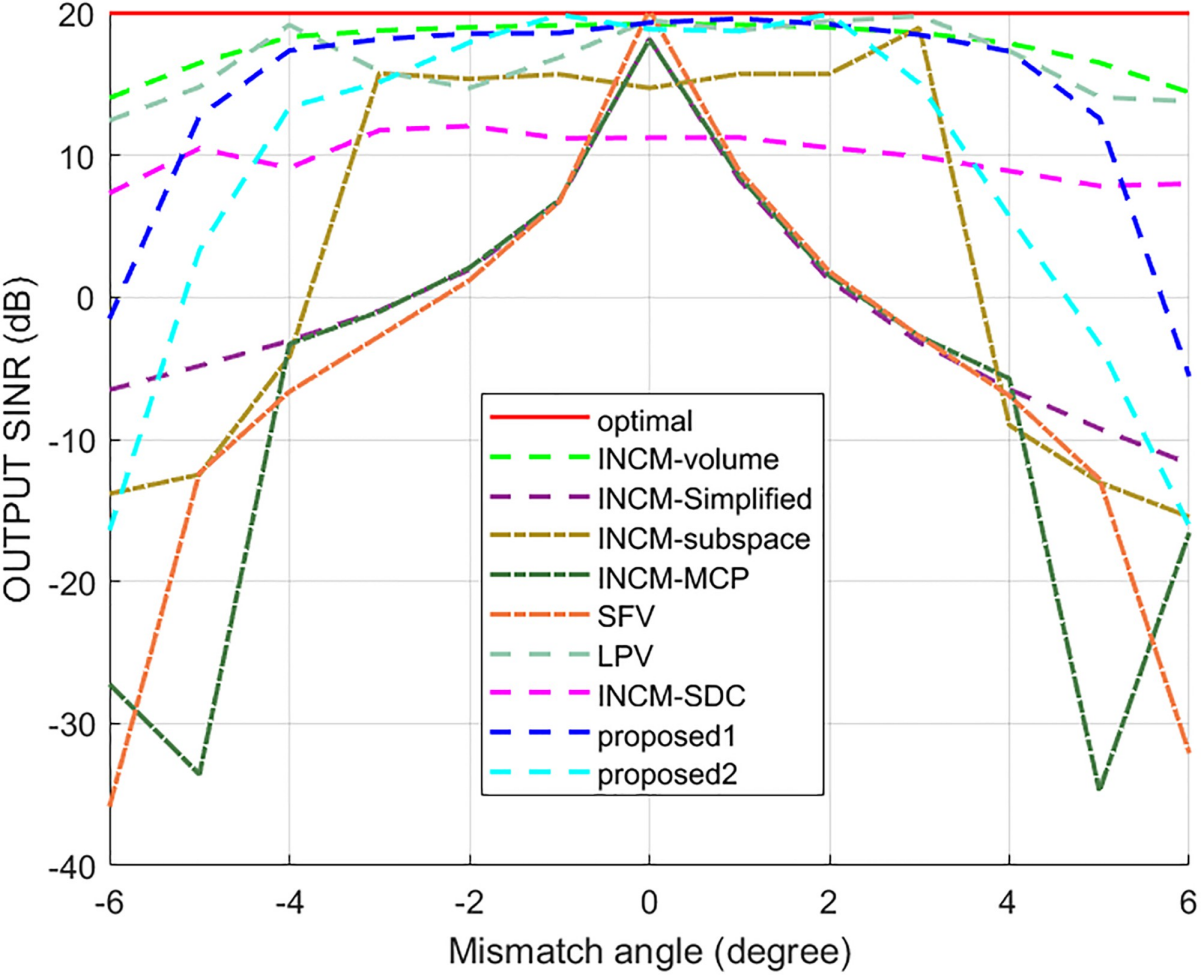
The output SINR versus the actual DOA.

In the second example, the effect of the signal’s look direction error on the output SINR was considered. It was assumed that the DOA estimation mismatches of all signals varied from −6° to 6°. The output SINR performances of the tested algorithms are shown in Fig 5. Although the SFV algorithm achieved the best results when the SV was specified, it was extremely susceptible to DOA mistakes. Similarly, the INCM-MCP and INCM-Simplified were also susceptible to the look direction error. The INCM-volume and proposed1 methods were not sensitive to the look direction error in the range of [−4°, 4°]. Because the INCM-volume and proposed1 methods collected more potential information about the SVs of the interferences during the ICM reconstruction process, they can work well under the mismatch caused by look direction error. In addition, the INCM-subspace and proposed2 methods were insensitive to the look direction error in the range of [−2°, 2°], but the INCM-subspace method performed worse than the INCM-volume, proposed1, and proposed2 methods. The LPV was insensitive to the look direction error in one incident direction, while the look direction error in the other directions affected its output to a certain extent.

### Mismatch caused by multiple model errors

**Fig 6:**
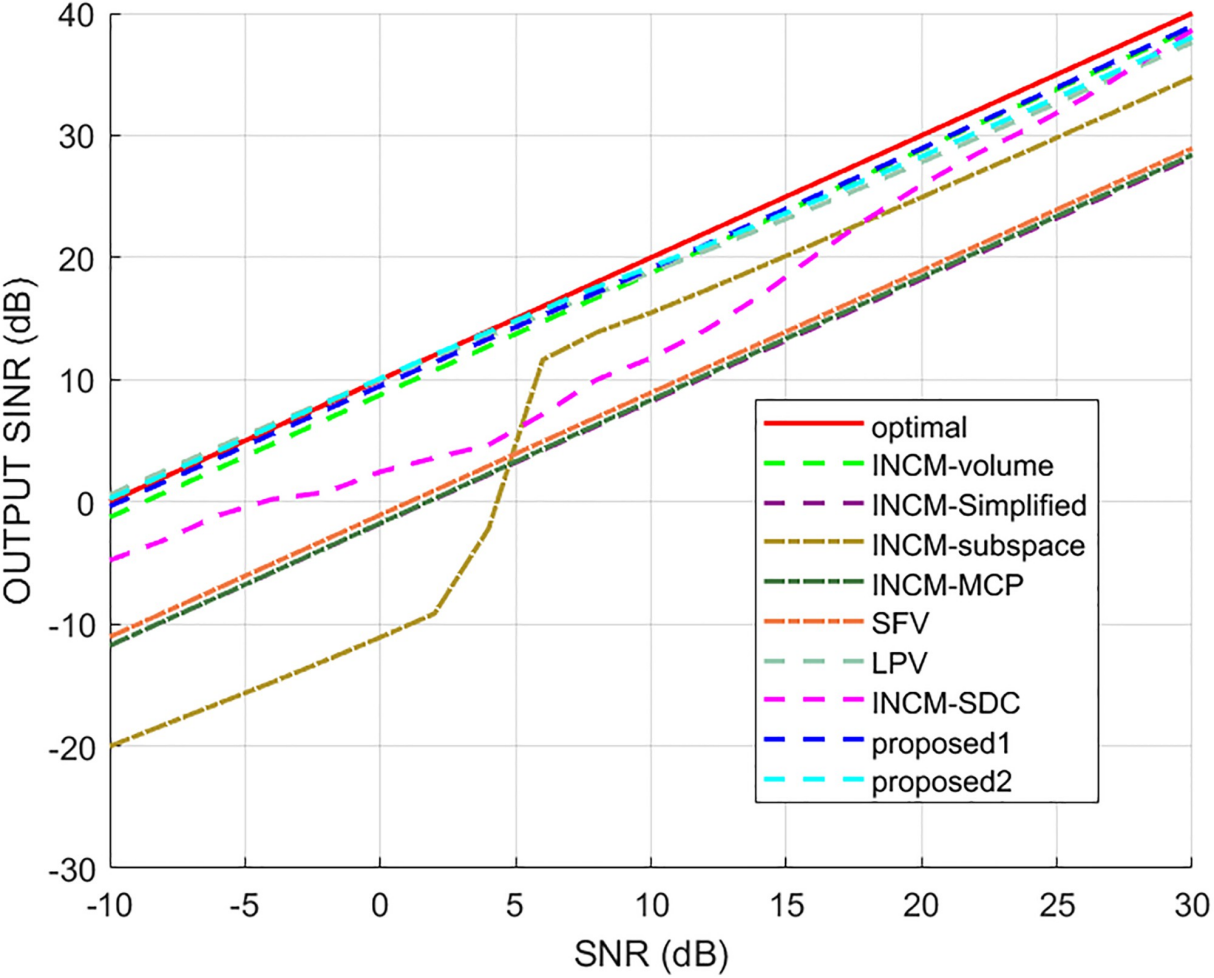
The output SINR versus the input SNR.

**Fig 7:**
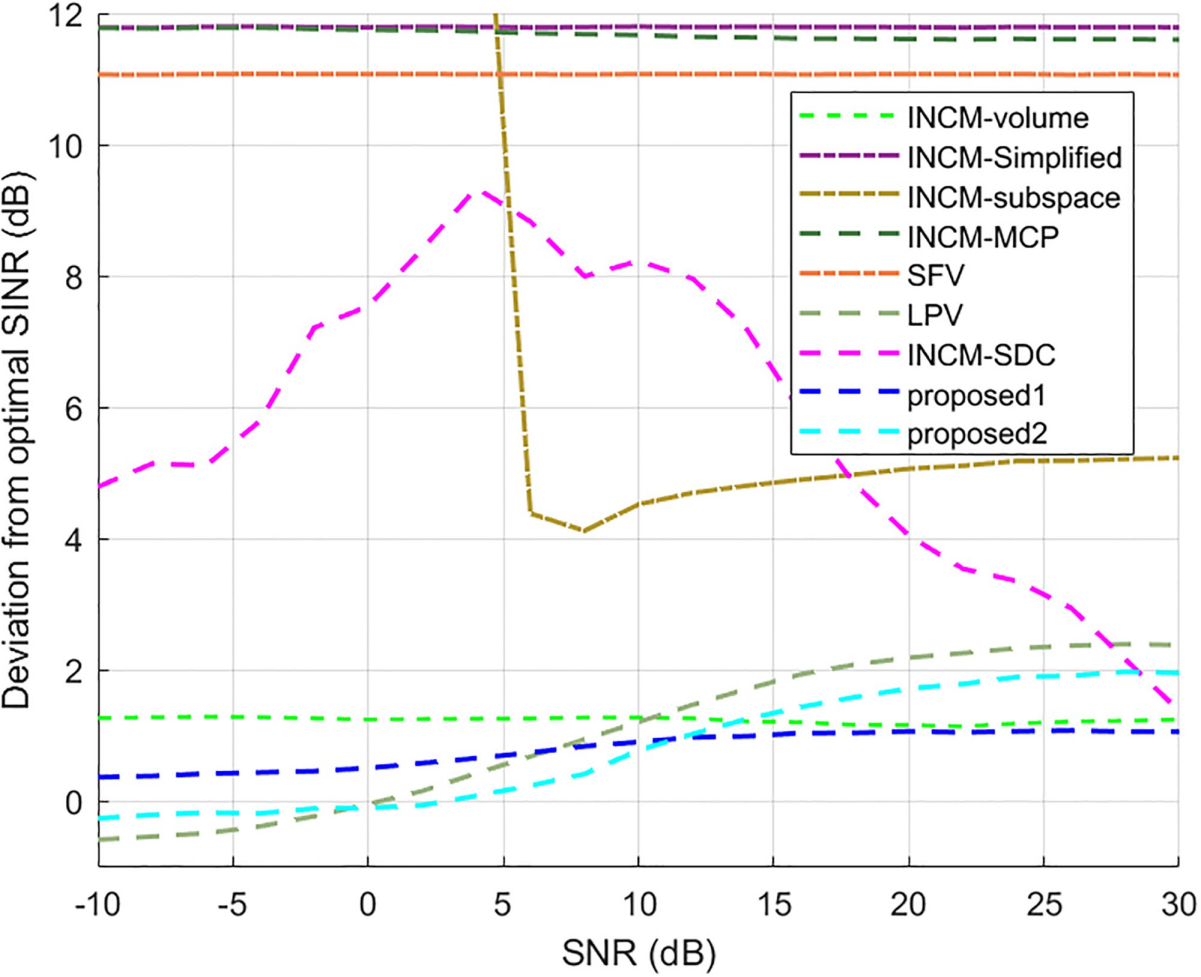
Deviations from the optimal SINR versus the SNR value.

**Fig 8:**
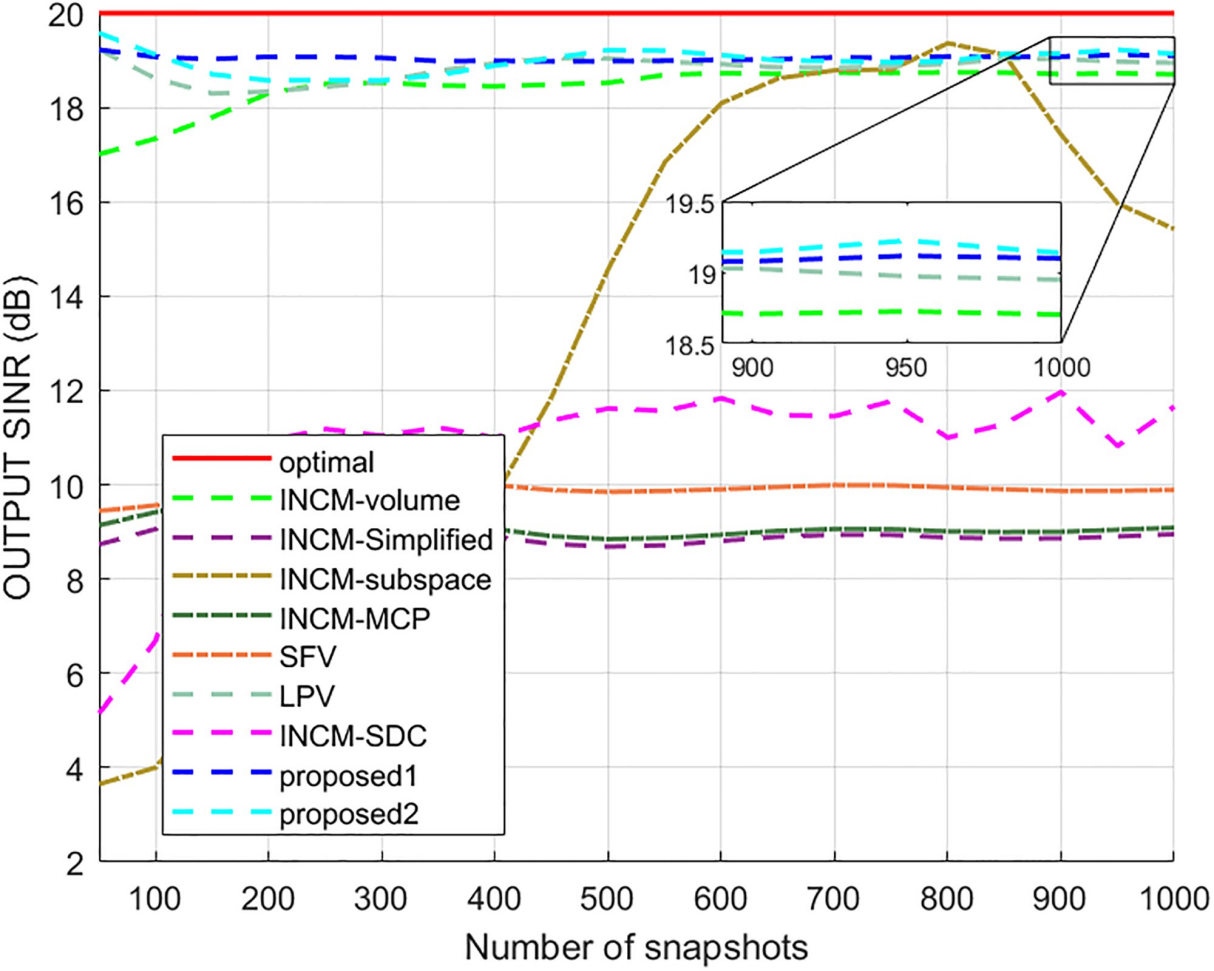
The output SINR versus the number of snapshots.

In this case, look direction error, gain error, phase error, and location perturbation were all considered. The look direction error was assumed to be uniformly distributed in [−2°, 2°]. The gain error and phase error were randomly distributed according to *N*(0, 0.05^2^) and *N*(0, 0.1*π*^2^), respectively, and the sensor location error was uniformly distributed in the interval [−0.05, 0.05] measured in sensor space. The mean output SINR versus the input SNR for a fixed number of snapshots is given in Fig 6. Fig 7 shows the deviation of each beamformer from the optimal beamformer. This shows that the proposed1 method performs well in the whole SNR range while the performance of the proposed2 method decreases when the input SNR increases. The output SINR versus the number of snapshots for fixed input SNR is given in Fig 8. The results for the proposed methods were close and better than the other techniques.

### Mismatch caused by coherent local scattering

In this scenario, the effect of a mismatch in the desired signal SV caused by coherent local scattering [40] on the array output was examined. Under this condition, the actual desired signal SV was expressed as $\text{a}=\text{a}\left(\theta_{1}\right)+\sum_{p=1}^{4}e^{j\varphi_{p}}\text{a}\left(\theta_{Lp}\right)$, where **a**(*θ*_1_) is the direct path, **a**(*θ*_*Lp*_) is the *p*th coherently scattered path in a direction *θ*_*Lp*_, and all *θ*_*Lp*_, *p* = 1, 2, 3, 4 are randomly distributed in a Gaussian distribution with mean of 5° and standard deviation of 1°; *φ*_*p*_ represents the path phase, whose value was independently and randomly generated from the interval of [0, 2*π*]. The look direction mismatch corresponding to the signals was uniformly distributed in the range of [−1°, 1°]. It should be noted that the look direction mismatch, *θ*_*Lp*_, and *φ*_*p*_ changed from run to run but were fixed from snapshot to snapshot.

**Fig 9:**
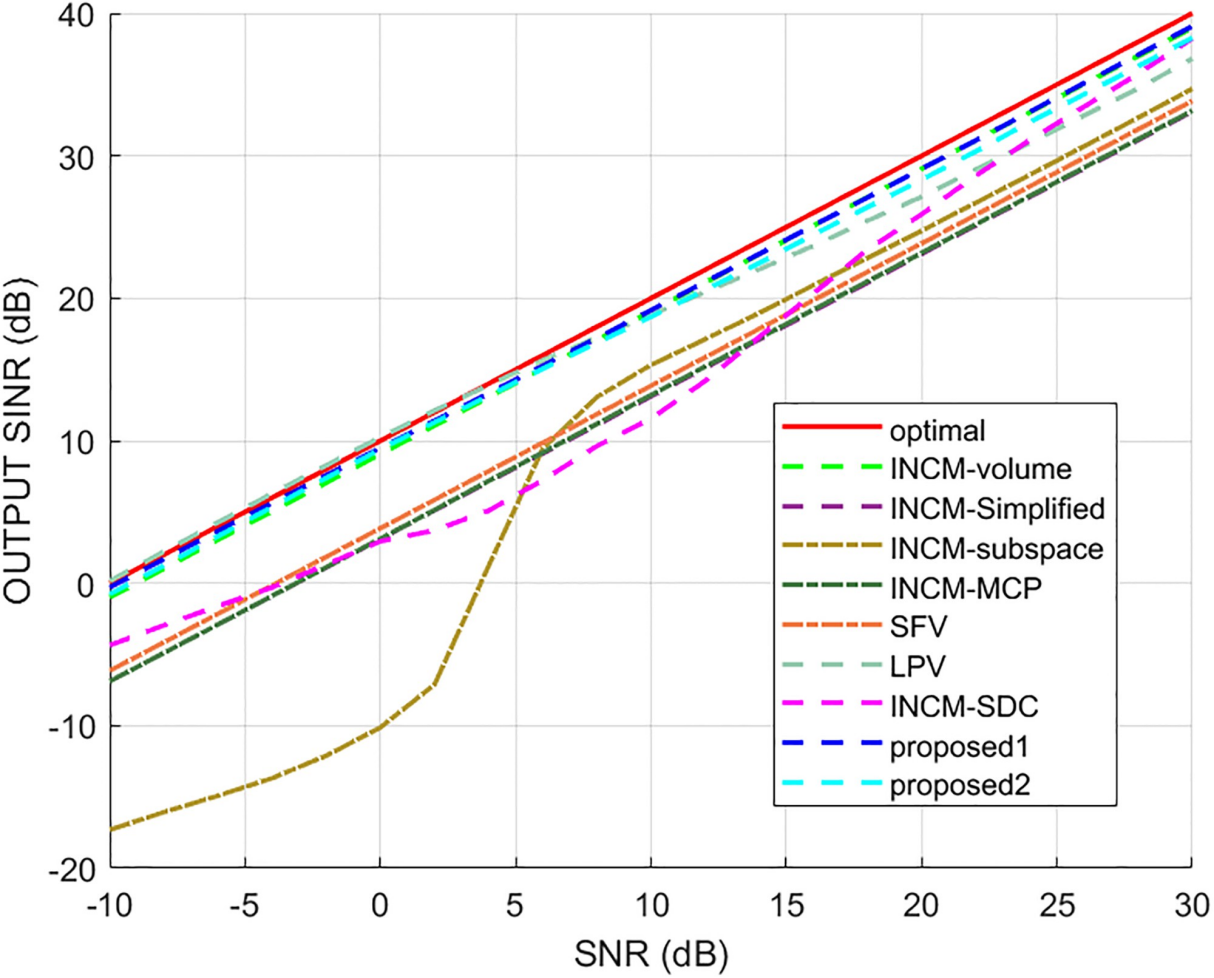
The output SINR versus the input SNR.

**Fig 10:**
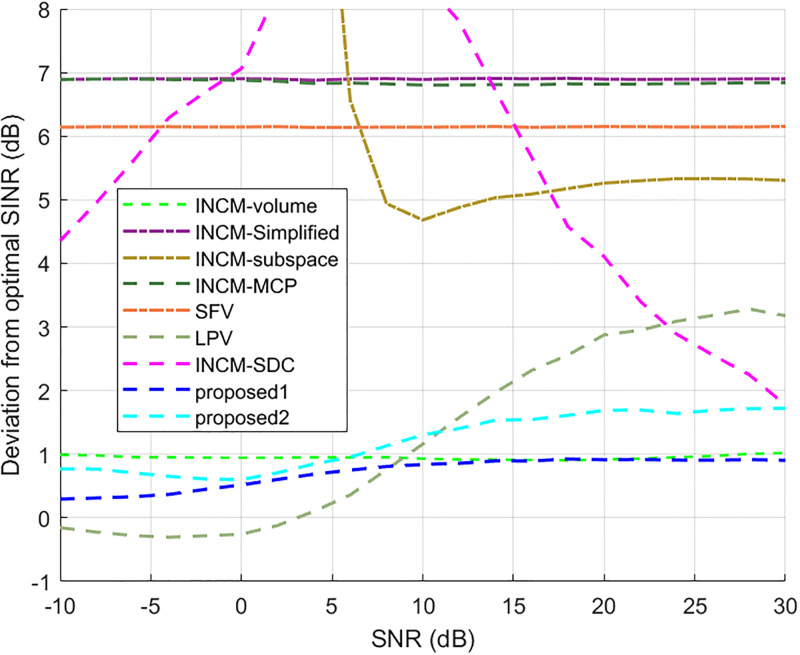
Deviations from the optimal SINR versus the SNR value.

**Fig 11:**
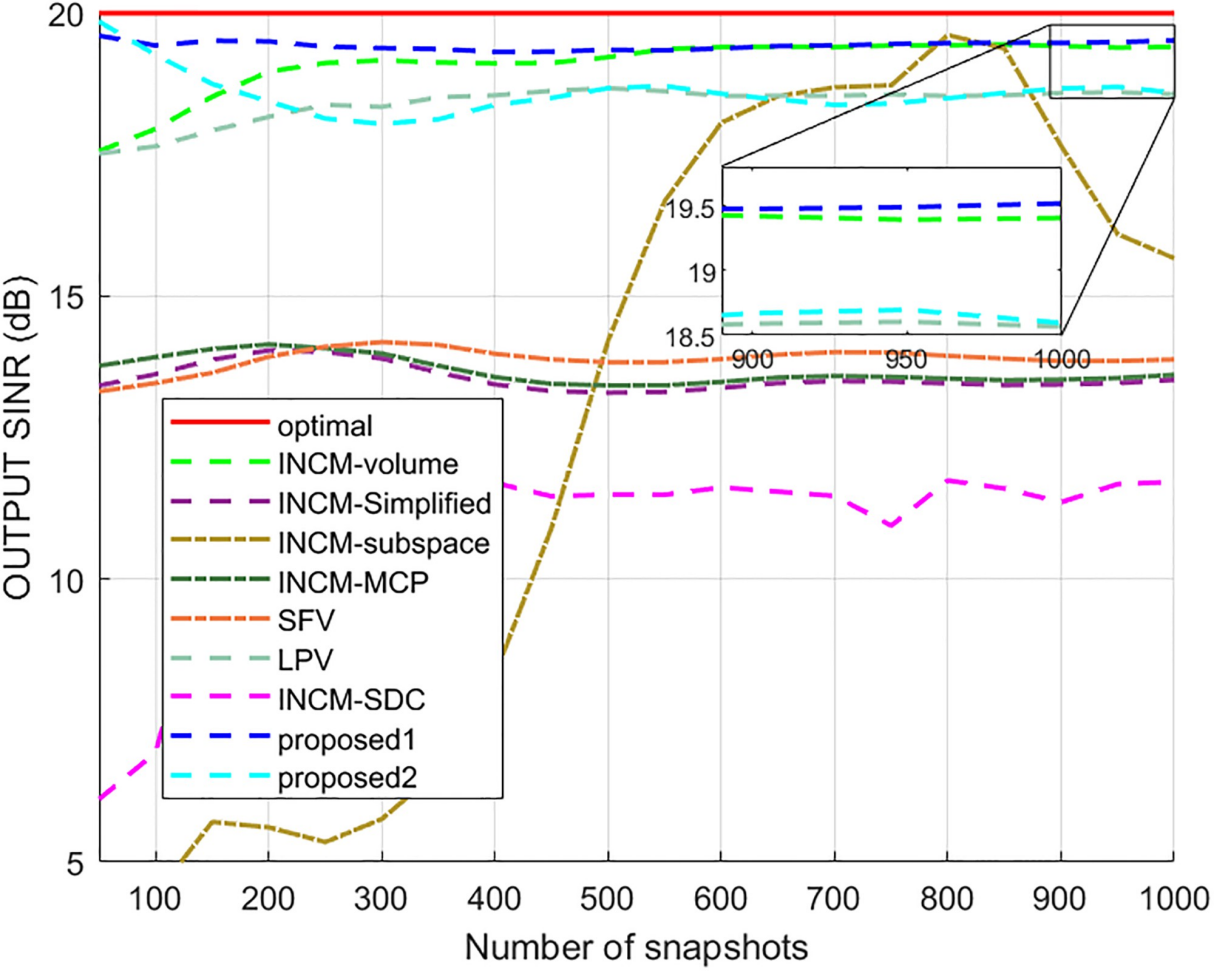
The output SINR versus the number of snapshots.

The output SINR results of all tested methods versus the input SNR are presented in Fig 9, and the deviations between them and the optimum result are presented in Fig 10. The results demonstrated that the proposed1 had the best performance among all methods, especially for the SNR values from −10 dB to 10 dB. This mismatch did not affect the results of the INCM-volume, proposed1, and proposed2 methods but had a negative effect on the results of the LPV, SFV, INCM-MCP, and INCM-Simplified methods. The output SINR results of all tested algorithms versus the number of snapshots are displayed in Fig 11. The results show that the proposed1 had the best performance among all methods for different numbers of snapshots, and the number of snapshots affected the output of the INCM-subspace method.

### Performance comparison for different arrays apertures

This experiment was conducted to analyze the performance of beamformers at different array apertures. The input SNR was fixed at 10 dB, the number of snapshots was set to 1, 000, and the input signals were unaltered. The look direction mismatch corresponding to the signals was uniformly distributed in the range of [−2°, 2°].

**Fig 12:**
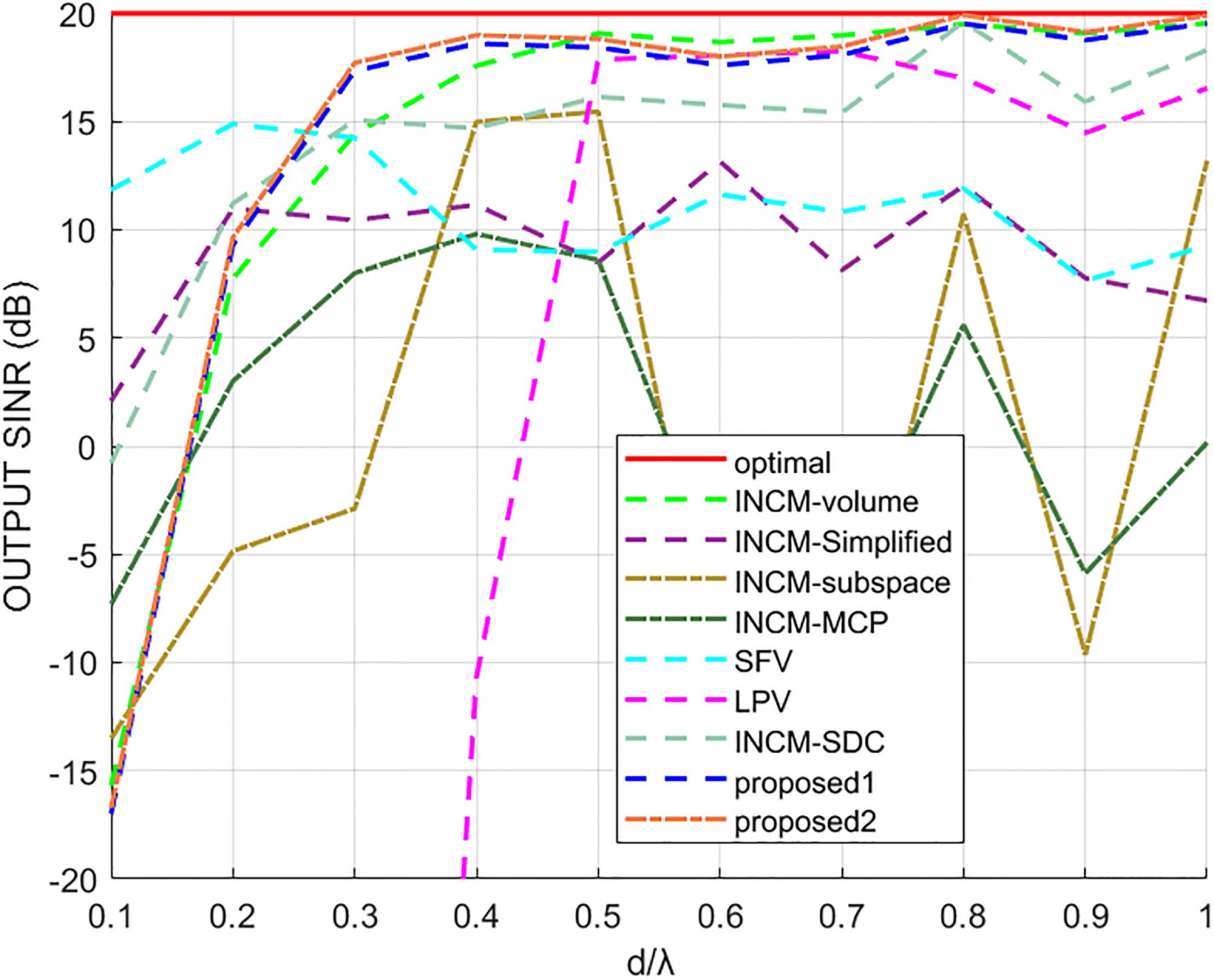
The output SINR versus the d/λ value.

First, the sensor number was fixed at 10, and it was examined how the test methods performed when the inter-sensor spacing was altered. The output SINR results of different methods versus the ratio of inter-sensor spacing *d* to signal wavelength λ are presented in Fig 12, where *d*/λ represents the ratio of array spacing to wavelength. The proposed1 and proposed2 methods could achieve stable performance when *d*/λ was higher than 0.3. While the output of the INCM-volume method and the LPV algorithm tended to be stable when *d*/λ was larger than 0.4, and the LPV algorithm was stable only when *d*/λ was from 0.5 to 0.7. The output SINR results of the other methods fluctuated with the value of *d*/λ.

**Fig 13:**
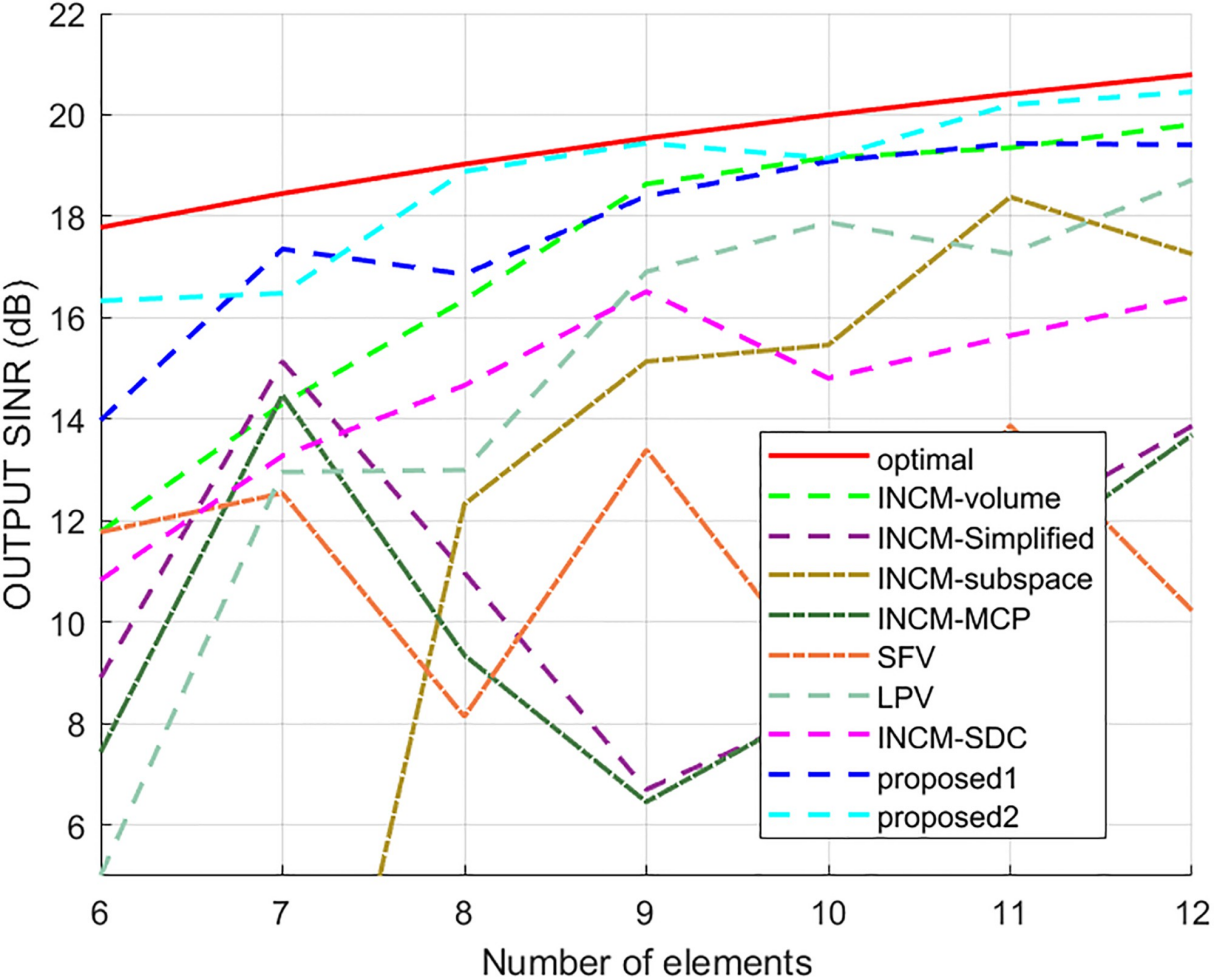
The output SINR versus the number of elements.

Next, the interval of elements was set to half wavelength to verify the output performance of the test algorithms for a different number of elements. The output SINR results of all tested algorithms versus the number of elements are presented in Fig 13, where it can be seen that the output results of the INCM-volume, proposed1, and proposed2 methods increased with the number of components. The proposed algorithms had the best performance among all methods when the number of array elements was less than nine.

### Performance comparison under extra degrees of freedom

The last example analyzed the beamforming performance under extra degrees of freedom (DOF). In this test, the number of input signals increased to 11, the sensor number was fixed to 10, and the spacing between array elements was half wavelength. The information on each input signal is shown in Table 1. It was supposed that the first signal was the desired signal.

**Table 1:** The settings of the input signals.

Input signal	1	2	3	4	5	6	7	8	9	10	11
Actual DOA (°)	5	-72	-60	-48	-35	-23	-10	20	40	53	65
Input SNR (dB)	/	10	30	10	10	30	30	10	20	30	20

**Fig 14:**
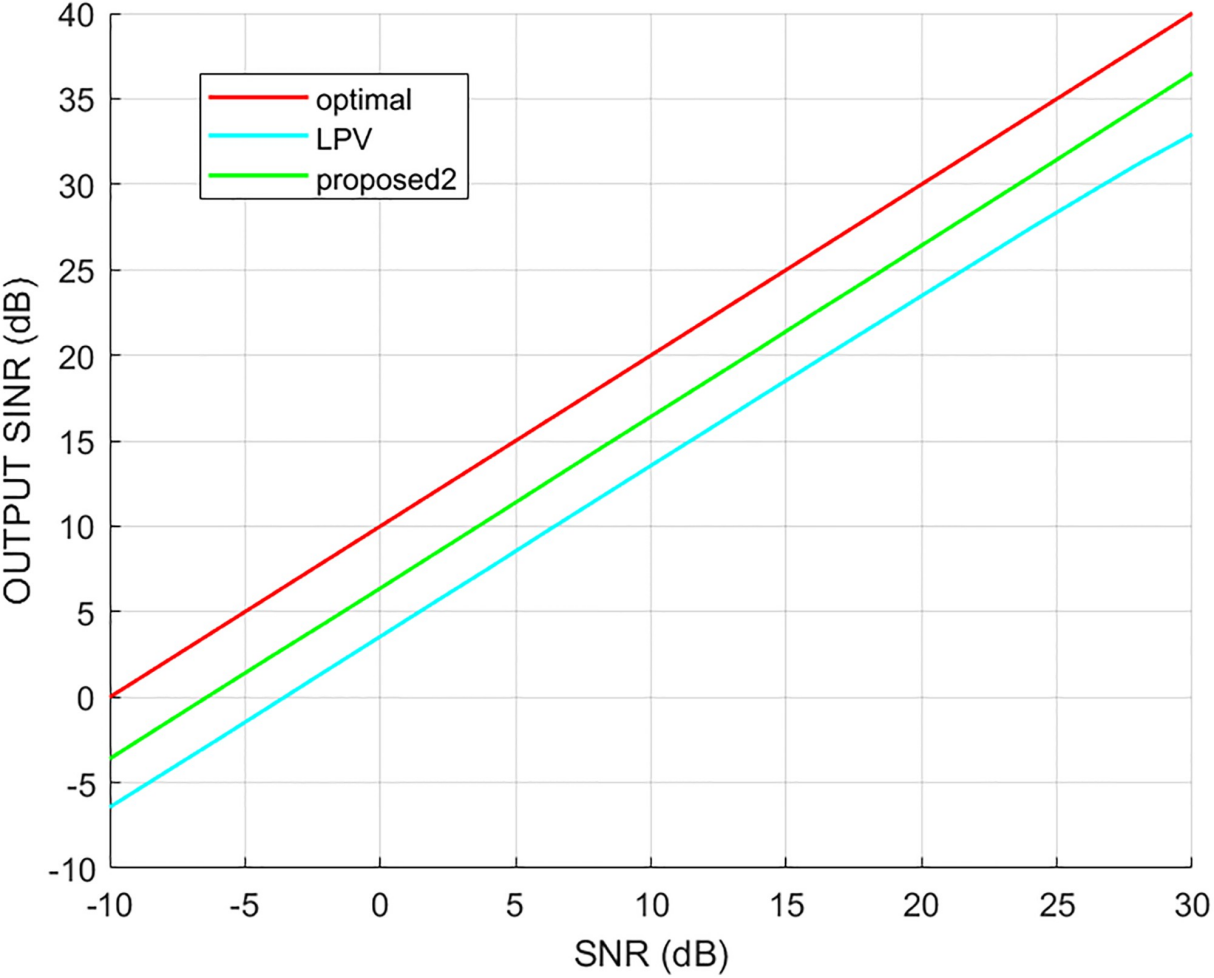
The output SINR versus the input SNR.

**Fig 15:**
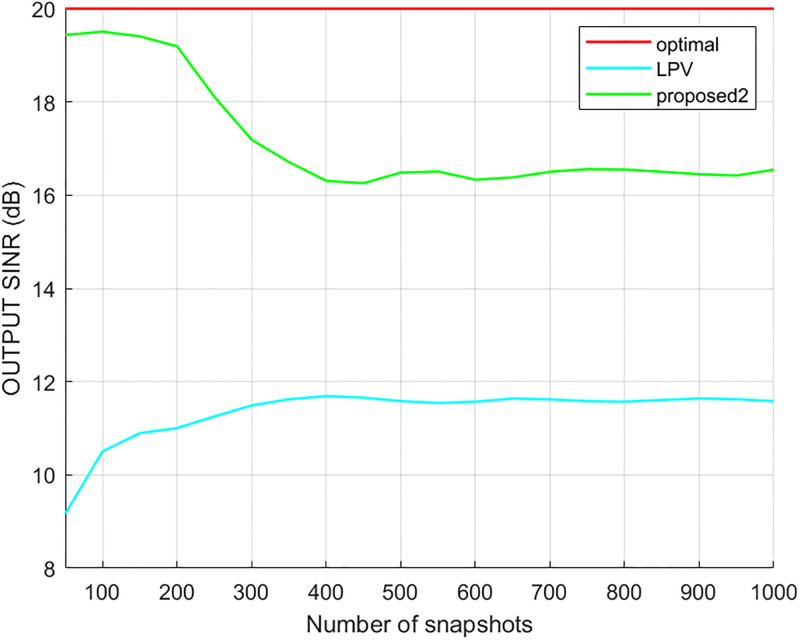
The output SINR versus the number of snapshots.

Since the LPV and proposed2 method are processed on the virtual extended array, both can realize the beamforming when the number of incident signals exceeds the number of array elements. The output SINR results of different methods versus the input SNR and the number of snapshots are shown in Figs 14 and 15, respectively, where it can be seen that the output of the proposed2 method was better than that of the LPV method. The LPV output results were stable when the number of snapshots was larger than 300. In addition, the output of the proposed2 method was better when the number of snapshots was less than 400 than when it was higher than 400.

## Conclusions

This paper proposes two RAB methods based on covariance matrix reconstruction denoted by proposed1 and proposed2. The proposed1 method performs angular sector integration and gradient vector integration to reconstruct the ICM and combines the estimated noise covariance matrix to reconstruct the INCM. Then the rebuilt ICM and INCM are used to optimize the desired signal’s SV. Simulation results demonstrate that this method can achieve good performance under different errors and a small array aperture by improving the robustness of the beamformers. The proposed2 method employs a virtual extended array and uses the conjugate property of a linear array. Data received at a virtual array element are produced by the linear prediction method, and the INCM of the extended array is reconstructed based on the ICM reconstruction algorithm similar to that used in the proposed1 method. The proposed2 method performs well for different array apertures and can increase the number of degrees of freedom of the beamformer by adding virtual elements. The complexity of the proposd2 method is higher than that of proposd1 due to the introduction of the extended array. To ensure beamformer robustness and consider computational efficiency, the proposed1 method should be considered when the number of signals is less than the number of array sensors. When the number of signals exceeds the array sensors, the proposed2 method should be considered. However, the output of the proposed2 method decreases as the look direction error increases. As a result, the robustness of the proposed2 method is an important issue to investigate in our future work.

## Supporting information

S1 Dataset (ZIP)
